# The Role of the Endothelin System in the Vascular Dysregulation Involved in Retinitis Pigmentosa

**DOI:** 10.1155/2015/405234

**Published:** 2015-11-03

**Authors:** Francesco Saverio Sorrentino, Claudio Bonifazzi, Paolo Perri

**Affiliations:** ^1^Department of Surgical Sciences, Unit of Ophthalmology, Ospedale Maggiore, Bologna, Italy; ^2^Department of Biomedical and Surgical Sciences, Section of Human Physiology, University of Ferrara, 44100 Ferrara, Italy; ^3^Department of Biomedical and Surgical Sciences, Division of Ophthalmology, University of Ferrara, 44100 Ferrara, Italy

## Abstract

Retinitis pigmentosa is a clinical and genetic group of inherited retinal disorders characterized by alterations of photoreceptors and retinal pigment epithelium leading to a progressive concentric visual field restriction, which may bring about severe central vision impairment. Haemodynamic studies in patients with retinitis pigmentosa have demonstrated ocular blood flow abnormalities both in retina-choroidal and in retroocular vascular system. Moreover, several investigations have studied the augmentation of endothelin-1 plasma levels systemically in the body and locally in the eye. This might account for vasoconstriction and ischemia, typical in vascular dysregulation syndrome, which can be considered an important factor of reduction of the ocular blood flow in subjects affected by retinitis pigmentosa.

## 1. Introduction

Recent studies have observed the reduced blood flow in both ocular and peripheral districts in patients suffering from retinitis pigmentosa (RP) [1]. The drop of the blood flow has been detected not only in retinal and choroidal vessels but also in retroocular vessels [2–4]. Also, a reduced baseline peak flow in the cutaneous capillary of fingers has been measured and a significantly longer recovery time has been found after cold provocation [4]. In the early stages of RP before detecting any ophthalmoscopic fundus modification, an increase of the arteriovenous passage time has been observed in the retina [5]. Using colour Doppler imaging, Cellini and coworkers demonstrated reduced peak systolic velocities in both ophthalmic arteries and posterior ciliary arteries [3, 4]. They also observed a certain augmentation of endothelin-1 (ET-1) plasma levels in patients with early-stage RP, even though some investigators took issue with this observation [6–8].

A sort of dysregulation of both blood supply and vessel caliber seems to occur often in RP patients. Konieczka has suggested that the primary vascular dysregulation syndrome, mainly characterized by an impaired vascular tone, might describe a wide range of local and systemic signs, such as ocular blood flow reduction with subsequent augmentation of ET-1 plasma levels in the eye and peripheral vasospasm in response to cold, emotional stress, or low blood pressure [1, 9–13]. Disturbed autoregulation of ocular perfusion provokes an irregular blood flow, which means an unstable retinal blood supply, and a sort of attenuation of retinal vessels with reduced neurovascular coupling [14, 15]. Consequently, both free radicals and oxidative stress substantially increase. Therefore, a large number of RP patients show a high prevalence of primary vascular dysregulation syndrome as the primary manifestation of ocular blood flow dysfunction [1]. Even if the exact pathophysiological mechanism is still unknown, the dysfunction of both autonomic nervous system and endothelial cells is currently investigated.

## 2. A Brief Overview of Retinitis Pigmentosa

RP is a clinical and genetic group of inherited retinal disorders. It is characterized by alterations of photoreceptors (PRs) and retinal pigment epithelium (RPE) resulting in progressive retinal degeneration. The most frequent symptoms are night-blindness and the growing impairment of visual field, perceived as tunnel vision, that may lead to legal blindness. Clinical signs found at ophthalmic fundus examination are characteristic: “bone-spicule” pigment deposits in the mid periphery along with RPE atrophy, attenuation of retinal vessels, waxy pallor of the optic disc, and relatively spared macula surrounded by a perimacular ring of depigmentation [16, 17].

The age of onset, the rate of progression, and the severity of RP are extremely variable depending not only on the genetic background but also on some influencing factors. Symptoms may start in childhood as well as in the early or mid-adulthood. Even though the progression of the disease is unpredictable, severe visual impairment typically occurs by the age of about 40–50 years. The prevalence of all different forms of RP is reported to be about 1 : 3500–5000 individuals and, nowadays, it is estimated that there are almost two million affected people around the world [18, 19].

The group of RP is characterized by a complex association between tremendous genotypic multiplicity and great phenotypic heterogeneity. The severity of the clinical manifestation depends on the penetrance of the disease gene, but also interactions between the gene expression and the environmental factors are of great importance [20].

## 3. The Ocular Blood Flow

The blood flow in the body is fine-tuned both systemically and locally. Specific biochemical and molecular signaling pathways keep monitoring the perfusion pressure, originally controlled by the heart pump, and the local resistances, depending on the vessel caliber. Vasospasm stands for inappropriate constriction of an artery [21]. In the presence of excessive vasoconstriction and/or altered vasodilation, the overall condition is described as vascular dysregulation and it is often accompanied by a barrier dysfunction. If this condition is associated with symptoms or signs, it is referred to as vascular dysregulation syndrome. It can affect any district in the body but mostly the eye [22].

The diameter of the vessels is regulated by contraction (vasoconstriction) or relaxation (vasodilation) of both smooth muscle cells, forming the arterial or venous wall, and pericytes, encircling the capillaries. The smooth muscle cells are very sensitive to modifications of tension (myogenic regulation), numerous metabolic factors originating from surrounding tissues, and biochemical signals coming from the autonomic nervous system (neurohumoral regulation) [23]. The local perfusion pressure, depending on the cardiac output, and the local resistance to flow, depending on the rate of local autoregulation, are the two main aspects of the tissue blood flow.

The endothelium is a very thin layer of cells lining vessels inwards. Beyond its function as mechanical barrier, it plays crucial roles in immune and inflammatory responses, in haemostasis, and in vascular tone regulation. Also it has autocrine, paracrine, and endocrine functions [24, 25].

Two vascular systems supply the eye: the choroidal and the retinal system. The first supplies the iris, the ciliary body, the choroid, and also, by diffusion, the outer retina, including the photoreceptors. The second entirely supplies the inner retina, comprising the retinal ganglion cells. The retinal vascular network is characterized by low perfusion rate, high vascular resistance, and high oxygen extraction. Conversely, the choroidal network shows a high perfusion rate, a low vascular resistance, and a low oxygen extraction. Just similar to the brain circulation, the ocular circulation is autoregulated. Autoregulation, actually effective within certain limits of perfusion pressure, is the intrinsic capacity to maintain constant flow despite changes in perfusion pressure [26].

The neurovascular coupling is the tight link between the caliber of retinal vessels and the neuroretinal activity. A signal from the central nervous system, closely connected to the retina, is able to evoke small changes in the retinal blood flow through complex pathways involving neurons, glia, and endothelial cells. Furthermore, the retinal vascular system features the blood retinal barrier which regulates the flux of ions, proteins, hormones, and water, even monitoring the infiltration of immune competent cells [22]. The choroidal vascular system is richly innervated [27]. It provides oxygen and other metabolites, controls eye temperature, and mostly contributes to the fine-tuning of accommodation by regulating choroidal thickness. This network, featuring fenestrated capillaries, is just partially autoregulated [22].

## 4. The Endothelin System

The endothelin (ET) system encompasses three active peptides (ET-1, ET-2, and ET-3), two G protein-coupled receptors (ET_A_ and ET_B_), and activating peptidases including the ET-converting enzymes (ECE-1 and ECE-2), [28]. Knock-out mice have been used as models to study pathophysiological role of the ET system. Some ET components are essential for the development of both tissues, such as cardiac or craniofacial tissue, and systems, such as enteric and nervous system [29–31]. Endothelin-1 (ET-1), widely distributed in human tissues, is produced by vascular endothelial cells and also by many other cells. Endothelin-2 (ET-2), with equally high affinities for both ET_A_ and ET_B_, is largely expressed in the gastrointestinal tract and it serves as local and paracrine/autocrine mediator [32]. Very little is known about the function of endothelin-3 (ET-3), except for the fact that it seems to be secreted somewhere near the relevant target cells, such as enteric neuroblasts expressing the ET_B_ [33].

First identified in 1988, ET-1 is the most powerful endogenous vasoconstrictor of both small and large vessels [34, 35]. It is a peptide with 21 amino acid residues and it is mostly released by endothelial cells of arteries, veins, and lymphatic vessels [36]. ET-1 acts as a modulator of the secretion of renin, vasopressin, and aldosterone, but at the same time it inhibits platelet aggregation [37]. Stressful conditions elicit increasing plasma levels of ET-1. Indeed, it has been observed in patients suffering with high blood pressure, arteriosclerosis, acute myocardial infarction, and diabetes mellitus [38–41]. Also hypoxic or oxidative stress, systemically and locally, has been identified as strong stimulus to raise primarily the levels of hypoxia-inducible factor 1 (HIF-1) and subsequently the levels of ET-1. In the eye, local synthesis and secretion of ET-1 are performed by many tissues, such as cornea, uveal tissue, retinal microvascular pericytes, RPE cells, and optic nerve, suggesting that the ET system could have an important role in the pathophysiology of some eye diseases [42–46]. Glaucoma, diabetic retinopathy, retinal vein/artery occlusion, proliferative vitreoretinopathy, and inherited retinal dystrophies are featured by impaired ocular blood circulation and they all present abnormal ET-1 plasma levels [47–51].

ET-2 is a protein encoded by the endothelin-2 gene (EDN2) and is a member of the ET system. It is referred to as macrophage chemoattractant and it is strongly induced in PRs in the course of retinal diseases and injury, being involved as stress signal to Müller cells through ET_B_ [52, 53]. Supposedly, a wide range of retinal disorders, including the inherited retinal dystrophies such as RP, converge on a relatively small number of molecular pathways going towards the cellular repair or death [54]. Müller cells, the most abundant glial cells in the retina, seem to monitor the status of retinal neurons, being generally activated after PRs' degeneration or death [55, 56]. There is evidence that injured or degenerated PRs start to release ET-2 stimulating the activation of the Müller cells fitted with ET_B_. These glial cells increase both the production of glial fibrillary acidic protein and the sensitivity to ET-2 by upregulating the ET_B_ as part of their repairing response [53, 57].

## 5. ET-1 Blood Levels in Retinitis Pigmentosa

ET-1 can be used as biochemical marker to assess ocular hemodynamics. To measure intraocular ET-1 blood concentration, colour Doppler imaging and laser Doppler flowmetry have been the two techniques specifically employed. Investigations showed the significant correlation between ET-1 plasma choroidal and plasma systemic concentrations [3]. In cases of RP, Cellini and coworkers have reported that a general increase in plasma levels of ET-1 elicits vasoconstriction both systemically and locally in the eye, causing retinal and choroidal hemodynamic impairment involving both ophthalmic artery and posterior ciliary arteries [2, 5, 6, 58, 59]. The role of the ET system in the pathogenesis of several ocular diseases, including the RP, is unquestionably important. However, at present there are some discordant studies about ET-1 plasma levels in people suffering from inherited retinal dystrophies. Cellini and colleagues have repeatedly observed high concentrations of ET-1 in these patients, whereas Ohguro and colleagues reported low levels of ET-1 [3, 8, 51, 60]. Regardless of their opposite experimental observations, common evidence is the decreased intraocular blood flow, initially in both choroid and optic nerve head and later in retina, due to abnormal plasma levels of ET-1 [61, 62].

A proper biochemical pathway responsible for the reduced intraocular blood flow is still not ascertained; however, there are some hypotheses under investigation. The first is about the migration of RPE cells to the inner layers of the retina after PRs' death. Local secretion of ET-1 from the proliferated and migrated RPE cells may stimulate repairing processes by deposition of extracellular matrix just underneath the retinal vessels [46]. As a consequence, thinning and fenestration of retinal vascular endothelial cells occur [63]. The second assumption considers the fact that the more the PRs die the less the ocular blood supply is required. Thus, a sort of vascular remodeling takes place in the chorioretinal tissue [64]. Another aspect to take into consideration is the low oxygen consumption by the degenerated and dying PRs. The subsequent vasoconstriction, mediated by increased levels of ET-1, is referred to as mechanism for limiting higher oxygen levels locally [65, 66].  Table 1 supplies a brief and clear summary of the crucial role of ET-1 in RP.

On the basis of recent investigations, in subjects with RP there is a statistically significant correlation between the augmentation of ET-1 plasma levels and the drop of the peak systolic velocity in both the ophthalmic artery and the posterior ciliary arteries [51]. The genetics are definitely remarkable, but the impaired retinochoroidal blood supply might be considered as an important modifier of the progressive loss of PRs. Furthermore, a sort of systemic dysfunction of the microcirculation seems to be in people with RP. In fact, Cellini and coworkers have measured, by means of laser Doppler flowmetry, a lower baseline cutaneous capillary blood flow and a longer warm recovery time in RP patients with respect to healthy subjects [3]. It has been observed that biochemical and metabolic alterations of the endothelial cells of retinal vessels and the augmentation of the intraocular oxygen concentration are triggering factors for the retinal production of ET-1. This molecule provokes high levels of intracellular Ca^2+^ in glial and neuronal cells and increases both neuronal activities and neuronal responses to glutamate [67]. Also, through ET_A_ receptors ET-1 may have a synergistic effect on the glutamate-induced neurotoxicity in the retina [68]. Other studies have also highlighted the pivotal role of ET-1 in relation to the apoptosis of the retinal ganglion cells through ET_B_ receptors [69].

## 6. Conclusions

To date, big efforts are still to be made to assess a fine correlation between genetic mutations and clinical manifestations in the RP. The most of mutations occur in genes coding for proteins involved in the cycle of vision, at the level of rods, cones, and RPE cells [1]. In the course of the disease, the PRs go towards apoptosis, so that the outer retinal nuclear layer flattens [70]. The pigment deposits, described as bone-spicule pigmentation, result from both RPE cell degeneration and migration into the neural retina after PRs' death [71]. There is evidence that oxidative stress and related conditions play an important role in the inherited retinal dystrophies [72]. Another important aspect of RP is the reduced blood flow in both ocular and peripheral districts along with a substantial increase in levels of inflammation [1, 22]. Strobbe and coworkers demonstrated a statistically significant correlation between ocular inflammation, detected by measuring aqueous flare with the noninvasive laser flare-cell meter, and high ET-1 plasma levels in patients suffering from RP [60]. This condition features alterations in choroidal thickness and blood flow leading to a boost in free radicals and oxidative stress.

Several general conditions, such as hypoxia, chronic oxidative stress, vascular dysregulation, or systemic inflammation, provoke the increase of ET-1 plasma levels. This molecule acts as powerful vasoconstrictor resulting in drop of the blood flow. This aspect is well-recognized in patients with RP who have low and impaired ocular blood flow, also characterized by progressive choroidal thinning and attenuation of retinal vessels. Some authors have observed that RP subjects feature an imbalance of the antioxidant-oxidant status in the peripheral blood [73]. Thus, the subclinical general inflammation and the substantial oxidative stress trigger the production of high levels of ET-1, which in turn bring about vascular dysregulation and diffuse hypoxic stress. This vicious circle contributes to activating and amplifying the inflammatory response. In the eye, vasospasm and altered intraocular perfusion bring about relative ischemia and, as a consequence, degeneration of the PRs [60].

Further investigations are needed to confirm the link between vascular dysregulation and subclinical inflammation in RP. However, in the next future research probably focuses on the development of novel drugs as antagonists of ET-1 and new antioxidants in order to better improve the vascular function systemically as well as locally.

## Figures and Tables

**Table 1:** The local and systemic increase of ET-1 and the vascular dysregulation syndrome in RP.

Drop in ocular blood flow	(i) Altered vessel caliber
	(ii) Impaired vascular tone
	(iii) Neurovascular coupling

Drop in O_2_ retinal supply	(i) Vasospasm
	(ii) Ischemia
	(iii) Hypoxia
	(iv) Biochemical and metabolic alterations of retinochoroidal endothelial cells

Degeneration of PRs	(i) Chronic oxidative stress
	(ii) Subclinical inflammation

ET-1: endothelin-1; RP: retinitis pigmentosa; PRs: photoreceptors.
